# Appropriate Management of Subcutaneous Tissue of Midline Abdominal Incisions

**DOI:** 10.7759/cureus.6549

**Published:** 2020-01-03

**Authors:** Nisreen Anfinan, Khalid H Sait

**Affiliations:** 1 Obstetrics and Gynecology, Faculty of Medicine, King Abdulaziz University, Jeddah, SAU

**Keywords:** surgical, wound, closure, drain, suture, infection, dehiscence

## Abstract

Objectives

To identify the optimal method for subcutaneous tissue management following midline abdominal incisions among patients with high thickness of subcutaneous fat (TSF).

Methods

A single-center prospective controlled trial among women undergoing elective gynecologic surgery by midline incision with TSF ≥ 3 cm. Incision was managed by suture approximation of Camper’s fascia (group 1), closed suction drainage (group 2), or no intervention (control). Groups were compared for the incidence of four-week postop wound complications including surgical site infection (SSI), superficial wound dehiscence (SWD), and seroma; and baseline and perioperative factors were analyzed using multivariate regression.

Results

Among 145 patients included (43.4% suture, 29.7% drain, 26.9% control), the overall incidence of wound complications was 15.2% (SSI 8.3%, SWD 0.7%, seroma 6.2%). The incidence of SSI was higher with suture (14.3%) versus drain (4.7%) and control (2.6%), while seroma was more frequent in drain (11.6%) versus suture (3.2%) and control (5.1%); however, both results were not statistically significant. Wound complication was independently associated with hemoglobin level (OR = 0.58, p = 0.019) and the occurrence of intraoperative complications (OR = 8.67, p = 0.048).

Conclusion

There is no statistical evidence about the optimal method of wound closure in the study population. Specific risk profiles can be constructed with an emphasis on preoperative anemia and intraoperative complications.

## Introduction

The development of postoperative negative outcomes as perceived either by the surgeon or by the patient, namely surgical wound complications, remained one of the significant threats to accomplish an optimal care of patients following surgeries [1]. Wound healing impairment occurs due to infection, necrosis of wound margins or adjacent soft tissue, seroma or hematoma; all of these complications have the potential to cause wound dehiscence. The latter may seem trivial, but it can be associated with devastating implications if not managed correctly. Instead of being a mild case that needs only local wound management and antibiotics, the presence of wound complications may cause serious sequelae with several reoperations and high rates of mortality. For example, surgical site infections following midline incisions were associated with a five-fold increased risk of reoperations, longer length of hospital stay, five times higher re-hospitalization rates, and a two-fold increase in the likelihood of mortality compared to those without complications and an additional cost of US$4,000 per wound [2,3].

Therefore, surgeons are required to mitigate the likelihood of wound complications via controlling the potential risk factors [4]. These could be related to the patient, surgical technique, or postoperative management. First, considering patient-related factors, the health and economic burdens of complications could increase with the global increase of obesity rates, which has been consistently associated with wound dehiscence and incisional hernia following laparotomies [5,6]. Second, the surgical technique has also a major role in reducing complication rates. More specifically, concerning midline incisions, evidence has shown that the quality of suture technique is paramount as revealed in the experimental and clinical studies [7]. Third, the success of wound healing using wound closure techniques may be challenging with increased thickness of subcutaneous fat (TSF) in obese patients [8]. This is because TSF was found to be a significant predictor for surgical site infections following midline laparostomy and colorectal surgeries [9,10]. In particular, studies demonstrated distinct TSF cut-off values that ranged between ≥20 mm and ≥30 mm for wound complications to develop [11,12].

In this context, this study was carried out to investigate the most appropriate methods for subcutaneous tissue management following midline vertical incisions among patients with low or high TSFs, by considering the occurrence of any surgical wound complication as the outcome of interest. Further, baseline demographic and clinical factors as well as perioperative factors were analyzed as cofactors of wound complication.

## Materials and methods

Population & setting

This prospective controlled trial was carried out at the Department of Obstetrics and Gynecology of King Abdulaziz University Hospital, Jeddah, Saudi Arabia, from March 2014 to October 2018. Women undergoing elective surgery by midline incision and having 3 cm or more subcutaneous tissue were included. All malignant and benign interventions were included. Patients who were hospitalized more than 24 hours prior to intervention and those with incision less than 3 cm depth were excluded. Patients presumed to be eligible were identified on admission and the allocated gynecological surgeon, among the three who participated to the study, obtained informed consent preoperatively. The study protocol was approved by the Unit of Biomedical Ethics, Faculty of Medicine, King Abdulaziz University.

Sampling & enrollment

A convenience sampling was used to include all eligible and consenting patients during the study period. According to the incision closure approach, included patients were allocated to one of the three groups: suture approximation of Camper’s fascia (group 1); closed suction drainage of the subcutaneous space (group 2); and no closure of the subcutaneous tissue (control group).

Procedures

Preoperative preparation was done as per the Department standard practice. All participants received preoperative antibiotic prophylaxis. Abdominal wall was cleaned with chlorhexidine-alcohol prepping solution without skin hair shaving. Surgical incision was made with the scalpel and hemostasis was undertaken by point cautery. The surgical procedure was performed through midline incision in the usual fashion, with the gynecological surgeon and his or her team. At the completion of the procedure, the pelvis and wound were both irrigated with warm normal saline solution. The fascia was closed in a mass fashion with a continuous #1 polydioxanone (PDS) suture. The depth of subcutaneous adipose tissue was measured with a disposable paper ruler from the fascia to the skin edge, half way between the umbilicus and pubis, to determine patient eligibility.

Each surgeon was assigned a group according to their preferred method of closure. The first surgeon used suture approximation of Camper’s fascia with running 2-0 Vicryl suture (group 1). The second one used suction drainage with 15 French round Jackson-Pratt (JP) drain of the subcutaneous space, which exited the wound through a separate stab incision; drains were upheld in place postoperatively, until output was less than 50 mL per 24 hours up to a maximum of four days (group 2). The third one preferred not to close the subcutaneous layer (control group). All of three surgeons used the same preferred suture closure method consistently for all included patients.

For all patients, the skin was re-approximated with staples, wounds were covered with non-adhesive gauze, and pressure dressing was applied, which remained in place until the morning of the second postoperative day. Staples were left in place for at least seven postoperative days. All patients were covered with prophylactic anti-coagulant with elastic stocking and received routine postoperative care at the discretion of the attending surgeon.

Data collection and follow-up

Demographic data included age, marital status, and parity. Baseline clinical data included height and weight with calculation of the body mass index (BMI), comorbidities (hypertension, diabetes, bronchial asthma, or other), and any previous surgery. These were analyzed as factors of wound complications.

Perioperative data were also recorded. In the preoperative time, data collected included type of diagnosis (benign or malignant), hemoglobin and albumin levels, as well as preoperative length of hospital stay (LOS). Intraoperative data included type of operation (total abdominal hysterectomy and bilateral salpingoophrectomy (TAHBSO) with or without staging, radical hysterectomy or debulking or myomectomy), any associated procedure, type of anesthesia, surgical time, intra-abdominal drain, occurrence of any complication, estimate blood loss (EBL), blood transfusion, subcutaneous depth and incision length, in addition to type of incision closure, etc.

All patients were evaluated daily during postoperative hospitalization and at two and six weeks postoperatively as an outpatient. Admission in intensive care unit (ICU), delayed feeding, total parenteral nutrition and the occurrence of any postoperative complication were recorded.

The outcome of interest consisted of the occurrence of any wound complication, which was divided into three categories: surgical site infection (SSI), superficial wound dehiscence (SWD), and seroma; by reference to clean wound.

Statistical methods

Statistical analysis was performed with the Statistical Package for Social Sciences (SPSS) for Windows version 21.0 (IBM Corp., Armonk, NY). The prevalence of wound complications was calculated with 95% confidence interval. Descriptive statistics were used to present baseline and perioperative factors of wound complication; categorical variables were presented as frequency and percentage, while numerical variables were presented as mean ± standard deviation (SD) for normally distributed variables and median (75th centile [P75]) for non-normally distributed ones. Analysis of factors associated with wound complication used chi-square test or Fisher’s exact test for categorical variables, as appropriate and independent t-test for normally distributed numerical variables. Multivariate binary logistic regression was carried out to analyze independent risk factors of wound complication; results were presented as odds-ratio (OR) with 95% CI. A p-value of <0.05 was considered to reject the null hypothesis.

## Results

Baseline demographic and clinical characteristics

A total 145 procedures were included; mean (SD) age of the patients was 51.83 (14.89) years, 77.9% were married, and 26.2% were nulliparous. Clinical data showed obesity (61.4%), hypertension (37.2%), diabetes (20.0%) bronchial asthma (5.5%), and previous surgery (38.6%). Of the patients, 44.8% were operated for malignant disease, and mean preoperative hemoglobin was 11.73 (1.54) mg/L (Table 1).

**Table 1:** Baseline demographic and clinical characteristics (N = 145) LOS: Length of stay; P75: 75th centile.

Parameter	Category	Frequency	Percentage
Age (years)	Mean, SD	51.83	14.89
Marital status	Single	16	11.0
	Married	113	77.9
	Divorced	7	4.8
	Widowed	9	6.2
Parity	0	38	26.2
	1-2	28	19.3
	3-5	45	31.0
	>5	34	23.4
Height (cm)	Mean, SD	153.72	7.31
Weight (kg)	Mean, SD	77.14	17.59
BMI (Kg/m^2^)	Mean, SD	32.24	7.34
BMI category (Kg/m^2^)	Normal (up to 24.9)	26	17.9
	Overweight (25.0-29.9)	30	20.7
	Class I obesity (30.0-34.9)	45	31.0
	Class II obesity (35.0-39.9)	21	14.5
	Class III obesity (≥40.0)	23	15.9
Comorbidities	Hypertension	54	37.2
	Diabetes	29	20.0
	Bronchial asthma	8	5.5
	Other	19	13.1
No. comorbidities	0	81	55.9
	1	30	20.7
	2	22	15.2
	3	12	8.3
Previous surgery	No	89	61.4
	Yes	56	38.6
Preoperative diagnosis	Malignant	65	44.8
	Benign	80	55.2
Preoperative hemoglobin level (mg/L)	Mean, SD	11.73	1.54
Preoperative albumin level (mg/dL)	Mean, SD	31.45	6.67
Preoperative LOS (days)	Median, P75	1.00	2.00

Procedure characteristics

Majority of patients underwent TAHBSO procedure (77.9%), and 55.2% had an associated procedure like omentectomy, lymphadenectomy and bowel resection include appendectomy. The most frequent type of anesthesia was general/epidural (58.6%) followed by general (35.2%). Surgical incision characteristics showed median (P75) subcutaneous depth (5.00 [5.50] cm), median (P75) incision length (20.00 [25.75] cm), and closure was carried out by suture (43.4%) or drain (29.7%), while nothing was applied in 26.9% of the cases. Other intraoperative data showed median surgical time (180.00 minutes), rates of blood transfusion (22.8%) and intraoperative complications (5.5%). Postoperative time was characterized by low incidence of ICU admissions (4.1%), delayed feeding (13.1%), total parenteral nutrition (1.4%), and postoperative complications (9.0%) (Table 2).

**Table 2:** Procedure characteristics TAHBSO: Total abdominal hysterectomy and bilateral salpingoophrectomy; EBL: Estimated blood loss; ICU: Intensive care unit; SSI: Surgical site infection; SWD: Superficial wound dehiscence; P75: 75th centile.

Parameter	Category	Frequency	Percentage
Intraoperative data			
Type of operation	TAHBSO	113	77.9
	TAHBSO and staging	9	6.2
	Radical Hysterectomy/debulking	19	13.1
	Myomectomy	4	2.8
Associated procedure	No	65	44.8
	Yes	80	55.2
Type of anesthesia	General/Epidural	85	58.6
	General	51	35.2
	Epidural	3	2.1
	Spinal	2	1.4
	General/Spinal	1	0.7
	Spinal/Epidural	1	0.7
Surgical time (min)	Median, P75	180.00	240.00
Frozen section	No	117	80.7
	Yes	28	19.3
Drain	No	97	66.9
	Yes	48	33.1
Intraoperative complications	No	137	94.5
	Yes	8	5.5
EBL (ml)	Median, P75	700.00	1000.00
Blood transfusion	No	112	77.2
	Yes	33	22.8
Subcutaneous depth (cm)	Median, P75; (rang = 2, 18)	5.00	5.50
Incision length (cm)	Median, P75; (range = 4.5, 41)	20.00	25.75
Incision closure	Suture	63	43.4
	Drain	43	29.7
	Nothing	39	26.9
Postoperative data			
ICU	No	139	95.9
	Yes	6	4.1
Delayed feeding	No	126	86.9
	Yes	19	13.1
Total parenteral nutrition	No	143	98.6
	Yes	2	1.4
Suprapubic catheter	No	94	64.8
	Yes	12	8.3
	Not specified	39	26.9
Postop. complications	No	132	91.0
	Yes	13	9.0
Postop. LOS (days)	Median, P75	4.00	6.00
Total LOS (days)	Median, P75	6.00	8.00

Wound outcome

The incidence of four-week wound complications was 15.2% (95% CI = 9.8%, 22.1%); including SSI (8.3%), SWD (0.7%) and seroma (6.2%). Distribution of wound complications by type of incision closure showed higher rate of SSI with suture (14.3%) compared to drain (4.7%) and control (2.6%), while seroma was more frequent with drain (11.6%) compared to suture (3.2%) and control (5.1%); however, both results did not reach statistical significance (Table 3).

**Table 3:** Wound outcome in overall cases and by type of incision closure Values are frequency (percentage), and percentages are calculated on column categories. SSI: Surgical site infection; SWD: Superficial wound dehiscence.

Outcome	Overall (N = 145)	Suture (N = 63)	Drain (N = 43)	Nothing (N = 39)	p-value
Clean	123 (84.8)	52 (82.5)	35 (81.4)	36 (92.3)	0.310
Any complication	22 (15.2)	11 (17.5)	8 (18.6)	3 (7.7)	
SSI	12 (8.3)	9 (14.3)	2 (4.7)	1 (2.6)	0.067
SWD	1 (0.7)	0 (0.0)	1 (2.3)	0 (0.0)	0.303
Seroma	9 (6.2)	2 (3.2)	5 (11.6)	2 (5.1)	0.198

Demographic and clinical factors associated with wound complication

The incidence of wound complications at four weeks postop was higher in patients with cancer (23.1% versus 8.8%) compared to their counterparts, respectively (p = 0.017) and was associated with low hemoglobin (mean [SD] = 10.99 [1.44] mg/L versus 11.87 [1.57] mg/L, p = 0.014) and albumin (mean [SD] = 28.30 [7.465] mg/dL versus 32.03 [6.38] mg/dL, p = 0.021) compared to patients without wound complications (Table 4).

**Table 4:** Baseline demographic and clinical factors associated with wound complication (N = 145) ^a^ Statistically significant difference; test used - t, independent t test; F: Fisher’s exact test, otherwise, chi square test was used.

Factor	Category	Wound complication				
		No		Yes		p-value
		N	%	N	%	
Age (years)	Mean, SD	50.97	14.58	56.64	16.04	0.100 t
Marital status	Single	13	81.3	3	18.8	0.964
	Married	96	85.0	17	15.0	
	Divorced	6	85.7	1	14.3	
	Widowed	8	88.9	1	11.1	
Nationality	Saudi	55	82.1	12	17.9	0.394
	Non-Saudi	68	87.2	10	12.8	
Parity	0	31	81.6	7	18.4	0.827
	1-2	23	82.1	5	17.9	
	3-5	39	86.7	6	13.3	
	>5	30	88.2	4	11.8	
BMI category (Kg/m^2^)	Normal	21	80.8	5	19.2	0.963
	Overweight	25	83.3	5	16.7	
	Class I obesity	39	86.7	6	13.3	
	Class II obesity	18	85.7	3	14.3	
	Class II obesity	20	87.0	3	13.0	
Hypertension	No	78	85.7	13	14.3	0.699
	Yes	45	83.3	9	16.7	
Diabetes	No	98	84.5	18	15.5	1.000^ F^
	Yes	25	86.2	4	13.8	
Bronchial asthma	No	117	85.4	20	14.6	0.349^ F^
	Yes	6	75.0	2	25.0	
Other	No	108	85.7	18	14.3	0.492^ F^
	Yes	15	78.9	4	21.1	
No. comorbidities	0	71	87.7	10	12.3	0.549
	1	23	76.7	7	23.3	
	2	19	86.4	3	13.6	
	3	10	83.3	2	16.7	
Previous surgery	No	74	83.1	15	16.9	0.477
	Yes	49	87.5	7	12.5	
Preoperative diagnosis	Cancer	50	76.9	15	23.1	0.017^a^
	Benign	73	91.3	7	8.8	
Preoperative hemoglobin level (mg/L)	Mean, SD	11.87	1.57	10.99	1.14	0.014^a^t
Preoperative albumin level (mg/dL)	Mean, SD	32.03	6.38	28.30	7.46	0.021^a^t
Preoperative LOS (days)	≤1 day	6	89.0	8	11.0	0.154
	>1 day	58	80.6	14	19.4	

Procedure-related factors of wound complication

Wound complications were more frequent in radical hysterectomy/debulking (36.8%) and TAHBSO + staging (22.2%), compared with TAHBSO (11.5%) and myomectomy (0.0%), and the difference is statistically significant (p = 0.027). Notably, increased subcutaneous depth (>5 cm) was associated with 27.0% incidence of wound complications, compared to 11.1% in case of incision depth ≤5 cm, and the difference was statistically significant (p = 0.020). However, no statistically significant association was found with incision length and the occurrence of wound complications (p = 0.241). Other intraoperative factors that were associated with wound complications included longer surgical time (26.9% in long [>180 min] versus 8.6% in short [≤180 min] interventions, p = 0.003), presence of intraoperative complications (50.0% in presence versus 13.1% in absence, p = 0.019), blood transfusion (26.4% versus 8.9%, p < 0.001). Further, the rate of wound complications was significantly associated with several postoperative factors such as ICU admission (p < 0.001), delayed feeding (p < 0.001), and postoperative complications (p < 0.001), etc. (Table 5).

**Table 5:** Procedure-related factors of wound complication (N = 145) EBL: Estimated blood loss; ICU: Intensive care unit; SSI: Surgical site infection; SWD: Superficial wound dehiscence; P75: 75th centile; ^a^ Statistically significant difference; test used - F: Fisher’s exact test, otherwise, chi square test was used.

Factor			Category	Wound complication				
				No		Yes		p-value
				N	%	N	%	
Intraoperative factors								
Type of operation	TAHBSO			100	88.5	13	11.5	0.027^a^
	TAHBSO + Staging			7	77.8	2	22.2	
	Radical hysterectomy/debulking			12	63.2	7	36.8	
	Myomectomy			4	100.0	0	0.0	
Associated procedure	No			59	90.8	6	9.2	0.072
	Yes			64	80.0	16	20.0	
Surgical time (min)	≤180			85	91.4	8	8.6	0.003^a^
	>180			38	73.1	14	26.9	
Frozen section	No			98	83.8	19	16.2	0.570^F^
	Yes			25	89.3	3	10.7	
Drain	No			83	85.6	14	14.4	0.724
	Yes			40	83.3	8	16.7	
Intraoperative complications	No			119	86.9	18	13.1	0.019^a^
	Yes			4	50.0	4	50.0	
EBL (ml)	≤700			71	88.8	9	11.3	0.144
	>700			52	80.0	13	20.0	
Blood transfusion	No			102	91.1	10	8.9	<0.001^a^
	Yes			21	63.6	12	26.4	
Subcutaneous depth (cm)	≤5			96	88.9	12	11.1	0.020^a^
	>5			27	73.0	10	27.0	
Incision length (cm)	≤20			67	88.2	9	11.8	0.241
	>20			56	81.2	13	18.8	
Incision closure	Suture			52	82.5	11	17.5	0.310
	Drain			35	81.4	8	18.6	
	Nothing			36	92.3	3	7.7	
Postoperative factors								
ICU admission		No		122	87.8	17	12.2	<0.001^a^
		Yes		1	16.7	5	83.3	
Delayed feeding		No		112	88.9	14	11.1	<0.001^a^
		Yes		11	57.9	8	42.1	
Total parenteral nutrition		No		123	86.0	20	14.0	0.022^a^
		Yes		0	0.0	2	100.0	
Suprapubic catheter		No		80	85.1	14	14.9	0.038^a F^
		Yes		7	58.3	5	41.7	
Postop. complications		No		118	89.4	14	10.0	<0.001^a^
		Yes		5	38.5	8	61.5	
Postop. LOS (days)		≤4		75	93.8	5	6.3	0.001^a F^
		>4		48	73.8	17	26.2	
Total LOS (days)		≤6		78	95.1	4	4.9	<0.00^a F^
		>6		45	71.4	18	28.6	

Baseline and preoperative predictors of wound complication

The multivariate model including all significant baseline and preoperative factors showed that only hemoglobin level (OR = 0.58, p = 0.019) and the occurrence of intraoperative complications (OR = 8.67, p = 0.048) were independently associated with wound complication (Table 6).

**Table 6:** Predictors of wound complication Binary logistic regression; dependent variable = occurrence of surgical wound complication. Ref: Category used as reference; ^a^ Statistically significant result (p < 0.05).

Factor	Category	OR	95% CI		p-value
Preoperative factors					
Diagnosis	Cancer	1.59	0.43	5.93	0.490
	Benign	Ref	-	-	-
Hemoglobin level	(mg/L)	0.58	0.36	0.91	0.019^a^
Albumin level	(mg/dL)	0.95	0.87	1.03	0.209
Intraoperative factors					
Type of operation	TAHBSO	Ref	-	-	0.540
	TAHBSO +Staging	6.01	0.47	75.95	0.166
	Radical Hysterectomy/debulking	1.73	0.38	7.92	0.481
	Myomectomy	-	-	-	0.999
Surgical time (min)	≤180	Ref	-	-	-
	>180	2.69	0.73	9.88	2.69
Intraoperative complications	No	Ref	-	-	-
	Yes	8.67	1.01	74.13	0.048^a^
Blood transfusion	No	Ref	-	-	-
	Yes	1.63	0.48	5.56	0.434
Subcutaneous depth (cm)	≤5	Ref	-	-	-
	>5	2.55	0.72	8.96	0.145

## Discussion

The results of this controlled trial showed no significant differences in the incidence of SSI, wound dehiscence, and seroma among all approaches of wound approximation. However, we identified several associated factors with wound complications, such as an established cancer diagnosis, preoperative decreased albumin levels, prolonged surgical time, blood transfusion, subcutaneous depth > 5 cm, and postoperative factors, including ICU admission, total parenteral nutrition, delayed feeding, prolonged LOS (>4 days) and postoperative complications. Importantly, low preoperative hemoglobin levels as well as the occurrence of intraoperative complications were independent risk factors of wound complications.

The results concerned with wound outcomes following different approximation techniques were conflicting. For example, a meta-analysis involving six randomized clinical trials (RCTs) has shown that suture closure in women with >2 cm subcutaneous fat led to reducing SWD by 34%, and this was potentially attributable to seroma prevention [13]. Nevertheless, recent studies showed variable outcomes. Dwivedi et al. randomized 60 patients equally to either suture approximation (polyglactin 910, 2-0) or suction syringe drainage and found that the rates of SSI, hospital readmission, and SWD were significantly higher with closed drainage management [14]. In contrast, Manoharan et al. assessed wound outcomes in 60 patients who underwent suture approximation or subcutaneous suction drains postoperatively [15]. The authors found beneficial effects of suctions drains since they reduced the incidence of SSI, SWD, and postoperative LOS when compared to primary skin closure.

However, other reports showed no significant differences between methods of postoperative wound management. In a large RCT conducted at an American gynecologic oncology department, Cardosi et al. revealed no differences in the rates of all wound complications, including SWD, cellulitis, seroma, hematoma, and abscess, following midline incisions in patients with ≥3 cm subcutaneous fat [16]. Another prospective RCT showed that the incidence rates of SSI were 7.7% and 5.7% when subcutaneous closed-suction drainage and no drainage were used, respectively, without statistically significant differences [17]. Failure to reach a consensus regarding the safest wound closure method may indicate differences in methodological designs, patient-related factors, the used tools/equipment, and surgeons’ expertise.

In the present study, preoperative anemia was independently associated with greater likelihood of postoperative wound complications. Patients with anemia may exhibit impaired tissue oxygenation, decreased collagen synthesis, and reduced capacity of neutrophil-mediated oxidative killing [18]. Additionally, these patients often require perioperative blood transfusion, which represents a confounding factor of inducing immunosuppression and can indirectly cause wound infection [19].

From another perspective, increased TSF (>5 cm) has been identified as an associated factor of wound complications in our study. Early and recent evidence indicated an important role of obesity in the development of postoperative SSI, and a TSF ≥ 3 cm was associated with 80% odd risk of developing a wound infection [20]. Several potential explanations have been postulated, such as reduced lymphocytic immunity, low oxygenation levels and decreased vascularity in the subcutaneous tissue, and impaired collagen synthesis in obese patients [21-23]. Interestingly, antibiotic administration is crucial and obese patients should be monitored regularly for possible complications since suboptimal antibiotic tissue penetration and dosage in subcutaneous tissue may be confounding factors that increase the risk of wound complications [24].

The present investigation covered a rare topic in obese women undergoing gynecologic surgeries via midline incisions. A prospective design was elected to assess the effects of confounding variables at our institution. However, our results should be cautiously interpreted due to some inherent limitations. Principally, failure to demonstrate statistical significance in the comparative analysis of wound complication incidence between the study groups may be due to reduced sample size in each group, entailing high type II error. Additionally, patients were allocated to each of the three groups based on surgeon’s preferences and hence there may be a risk of selection bias.

## Conclusions

There is no statistical evidence about the optimal and safest method of wound closure in obese women undergoing gynecologic surgeries. Patients with TSF of at least 3 cm should be considered and adequately monitored for the prevention of wound complications, particularly clinically vulnerable patients, such as those with preoperative anemia, malignancy, and hypoalbuminemia. Complicated cases during and after the operations should be thoroughly checked for infectious and mechanical wound disruptions. Notably, specific risk profiles can be constructed for each individual patient to identify patients at high risks of further complications, with an emphasis on preoperative anemia and intraoperative complications. Antibiotic prophylaxis, which was not investigated in the present study, may be useful to prevent SSI and both indication and dosage should be adjusted based on body weight profiles.
